# Accuracy of Commonly-Used Imaging Modalities in Assessing Left Atrial Appendage for Interventional Closure: Review Article

**DOI:** 10.3390/jcm7110441

**Published:** 2018-11-14

**Authors:** Ramez Morcos, Haider Al Taii, Priya Bansal, Joel Casale, Rupesh Manam, Vikram Patel, Anthony Cioci, Michael Kucharik, Arjun Malhotra, Brijeshwar Maini

**Affiliations:** 1 Department of Internal Medicine, Florida Atlantic University, Boca Raton, FL 33431, USA; morcos.ramez@gmail.com (R.M.); jcasale3@health.fau.edu (J.C.); rmanam@health.fau.edu (R.M.); patelv@health.fau.edu (V.P.); 2 Department of Cardiovascular Diseases, Florida Atlantic University, Boca Raton, FL 33431, USA; haltaii@health.fau.edu (H.A.T.); Pbansal@health.fau.edu (P.B.); 3 College of Medicine, Florida Atlantic University, Boca Raton, FL 33431, USA; acioci@health.fau.edu (A.C.); mkucharik2016@health.fau.edu (M.K.); 4 University of Miami, Coral Gables, FL 33124, USA; a.malhotra2@umiami.edu; 5 Tenet Florida & Department of Cardiovascular Diseases, Florida Atlantic University, Boca Raton, FL 33431, USA

**Keywords:** left atrial appendage, WATCHMAN occlusive device, 2D transesophageal echocardiography, 3D transesophageal echocardiography, computerized tomography

## Abstract

Periprocedural imaging assessment for percutaneous Left Atrial Appendage (LAA) transcatheter occlusion can be obtained by utilizing different imaging modalities including fluoroscopy, magnetic resonance imaging (MRI), computed tomography (CT), and ultrasound imaging. Given the complex and variable morphology of the left atrial appendage, it is crucial to obtain the most accurate LAA dimensions to prevent intra-procedural device changes, recapture maneuvers, and prolonged procedure time. We therefore sought to examine the accuracy of the most commonly utilized imaging modalities in LAA occlusion. Institutional Review Board (IRB) approval was waived as we only reviewed published data. By utilizing PUBMED which is an integrated online website to list the published literature based on its relevance, we retrieved thirty-two articles on the accuracy of most commonly used imaging modalities for pre-procedural assessment of the left atrial appendage morphology, namely, two-dimensional transesophageal echocardiography, three-dimensional transesophageal echocardiography, computed tomography, and three-dimensional printing. There is strong evidence that real-time three-dimensional transesophageal echocardiography is more accurate than two-dimensional transesophageal echocardiography. Three-dimensional computed tomography has recently emerged as an imaging modality and it showed exceptional accuracy when merged with three-dimensional printing technology. However, real time three-dimensional transesophageal echocardiography may be considered the preferred imaging modality as it can provide accurate measurements without requiring radiation exposure or contrast administration. We will present the most common imaging modality used for LAA assessment and will provide an algorithmic approach including preprocedural, periprocedural, intraprocedural, and postprocedural.

## 1. Introduction

Atrial Fibrillation (AF) is a major burden on public health, it is estimated to be the cause of ≥15% of all strokes in the United States, and >100,000–125,000 embolic strokes per year, of which >20% are fatal [1]. AF risk factors, pathogenesis, prevention, and treatment are beyond the scope of this review. We will focus on the classification when patients may or may not have valvular heart disease. The distinction still an area of debate. Valvular AF is the terminology used in those patients who have heart valve disorder or a prosthetic heart valve. Nonvalvular AF is generally referred in those patients who have other etiology causing the AF. The rapid and chaotic heartbeats restrict the left atrium from pumping the blood properly, which may cause it to pool and form a clot. More than 90% of thrombi in AF is formed in the left atrial appendage (LAA) [2]. The standard treatment for AF is heart rate or rhythm control and stroke prevention. Prophylactic anticoagulation is the gold standard to prevent embolic strokes in AF patients with CHADS-VASC score greater than or equal to two. For many years there has been no alternative treatment available to prevent strokes in AF patients who have high risk of bleeding and only 50–60% are therapeutically anticoagulated which make the effective long-term anticoagulation very challenging [3].

In 2001 a successful percutaneous implantation of a device to occlude the LAA cavity was done in a patient with non-valvular AF to prevent embolism [4]. Interventional closure of the LAA employing the Watchman device (Boston Scientific) was shown to be non-inferior to Oral anticoagulation (OAC) in randomized trials and has since been approved in the United States and Europe [5]. The LAA exhibits complex anatomy that commonly varies morphologically among different individuals. Post-mortem analysis of 100 left atrial appendages has demonstrated significant variability in appendage shape, dimensions, and the number of lobes presents [6]. Therefore, accurate visualization of the LAA and appreciation of its morphological considerations is an essential step in occlusion procedures. Specifically, accurately measuring the dimensions of the LAA ostium, landing zone, and maximum length of the main anchoring lobe is necessary for selecting an adequately sized occlusion device successful device placement [4]. Choosing a device that is too small increases the risk of device instability and peri-device leakage, whereas selecting a device that is too large increases the risk of LAA perforation and cardiac tamponade [7,8]. Additionally, improper device selection can result in intra-procedural device changes and recapture maneuvers and increasing length of the procedure [9].

We present a review on the most commonly used imaging modality for pre-procedural planning and assessment of the LAA morphology, which include 2D Transesophageal echocardiography (2D TEE), 3D Transesophageal echocardiography (3D TEE), Computed tomography (CT), and 3D Printing (3DP).

### 1.1. 3D TEE Modality Is Superior to 2D TEE

2D TEE has been the most commonly used imaging modality for pre-procedural planning. However, three-dimensional multiplanar transesophageal electrocardiography is a more accurate alternative to 2D TEE in the assessment of the LAA morphology. Advantages of 3D TEE vs. 2D TEE are illustrated in (Table 1), and comparative studies using 3D TEE are illustrated in (Table 2).

Zhou et al. found 3D TEE to be more accurate than 2D TEE for measuring the LAA Landing zone, LAA depth, and LAA ostial dimensions, LAA morphology after the occlusion device deployment, and visualizing any residual shunts around the entire device in one more view. In the Zhou et al. study, a residual shunt of less than 1mm was identified in three cases by 3D TEE and only once by 2D TEE [7]. Salzman et al. determined that area-derived diameter (ADD) and perimeter-derived diameter (PDD) measurements obtained via 3D TEE correlated well with the occlusion device size chosen in the procedure. Furthermore, the 3D landing zone measurements demonstrated a higher reproducibility relative to 2D TEE [8].

Yosefi et al. found that RT3DTEE provides more accurate measurements of the maximal LAA orifice than 2D TEE. 2D TEE significantly undersized the diameter of the LAA orifice relative to RT3DTEE, when compared to CT [10]. Nucifora et al. found that RT3DTEE is in more significant agreement with the dimensions obtained from CT as demonstrated by smaller bias and narrower limits of agreement with CT. Therefore, these authors believe that RT3DTEE may be the preferred imaging modality to assess LAA dimensions as it can provide accurate measurements of the LAA without requiring radiation exposure or contrast administration [11].

The real-time 3DTEE method is a feasible, fast way to assess the LAA number of lobes, the area of the orifice, maximal LAA diameter, minimum LAA diameter, and LAA depth with similar accuracy to RT3DTEE and CT according to a study published by Yosefy et al. Real-time 3DTEE consists of converting a 3DTEE image into three 2D planes (X,Y,Z), at which time a 360 degree rotational in the sagittal plane creates a single “stop shop” image that displays all aspects of the LAA morphology including number of lobes, orifice area, and maximal and minimal diameter [12]. Nakajima et al. determined that 3D TEE could accurately visualize LAA morphological variations. They studied 55 patients in normal sinus rhythm and 52 patients with atrial fibrillation. 3D TEE provides adequate 3D full volume images of all patients in NSR, whereas sufficient images were obtained in 94.6% of patients with AF using zoom mode. Excellent correlation was found between full volume mode and zoom mode [13].

### 1.2. CT Is More Accurate Than TEE

CT has been considered the gold standard for visualizing the LAA for its ability to acquire 3D volumetric data of the LAA at various points in the cardiac cycle [4]. However, it has only recently emerged as an imaging modality for sizing the LAA before occlusion and for post-procedural evaluation of residual peri-device shunts. Although TEE remains the most commonly used imaging modality for sizing the LAA, CT may be the most accurate imaging modality. Yosefy et al. compared 2D TEE to CT and found 2D TEE to be non-inferior to CT for determining LAA area and volume. Additionally, Yosefy et al. found that of 30 patients who underwent routine TEE examination and CT in the workup of PE, RT3DTEE was found to yield measurements not significantly different than CT for the number of LAA lobes, LAA depth, LAA internal area, and LAA maximal and minimal diameter. They concluded that 3DTEE might be more practical for sizing the LAA due to its accuracy, lack of radiation, and bedside capabilities [10]. In contrast, other studies have demonstrated that measurements obtained via TEE and CT are not interchangeable and may result in clinically significant consequences. One such study by Sievert et al. showed that LAA sizing by 2D TEE alone might result in the selection of a closure device that is undersized by 20–40% [2]. Advantages of CT are illustrated in (Table 3), and comparative studies using CT are illustrated in (Table 4).

MSCT is a more accurate tool in selecting proper LAA closure device size than the conventionally used TEE according to the study by Chow et al. [14] 2D-TEE measurements of orifice size are not interchangeable with those obtained via CT according to a study by Rawjani et al. The researchers concluded that due to the irregular and eccentric nature of the LAA orifice, obtaining mean orifice diameter measurements may be more accurate than planar maximal diameters for sizing circular occluder devices [15].

In the study by Wang et al. patients who underwent advanced CT imaging at this site required 1.245 devices per implantation attempt with 100% success rate, compared to patients in the first half of the PROTECT AF study who averaged 1.8 devices used per implantation attempt with an 82% success rate. Accurate sizing of the LAA landing zone is critical in successful implantation of the WATCHMAN, and this study suggests high-resolution volumetric imaging with CT should be preferred over TEE [9].

Budge et al. determined that measurements obtained via CTsb, CTp, and TEE are not interchangeable. CTsb was found to yield larger mean orifice diameters than both TEE and CTp, which produced similar mean orifice diameters. It was speculated that this was due to foreshortening associated with 2D modalities. Furthermore, when compared to TEE, both CT modalities yielded larger ostial measurements for small LAA orifices and smaller ostial measurements for larger LAA orifices within this cohort [16].

### 1.3. The Use of 3D Printing Can Facilitate LAA Occlusion

As more LAA occlusion procedures have been conducted, physicians have recognized the unique and diverse morphology of the LAA [17]. This anatomical intricacy may be deceptively portrayed in standardized diagnostic modalities such as 2D and 3D transesophageal echocardiography (TEE), which are the conventional pre-procedural image technique. 3D CT characterization may provide exceptional accuracy when merged with 3D printing technology. By creating a model customized to each patient’s anatomy, a physical Watchman device (Boston Scientific, Marlborough, MA, USA) can be implanted ex vivo so that spatial navigation and geographic accuracy of the left atrium may be established before the cardiac catheterization procedure commences. The following findings show this modality technology applied and successively replicated. They also suggest 3D CT as the best imaging technique when establishing device size. Hell et al. [18] and Li et al. [19] supported the use of 3D printing while, Goiten et al. did not support it [20] (Table 5).

Hell et al. and Li et al. in prospective studies both found that 3D printing of LAA was a feasible mechanism of predicting correct Watchman devices. In the study conducted by Hell et al. Mean LAA ostium diameter based on TEE was 22 ± 4 mm and based on CT 25 ± 3 mm (*p* = 0.014) [18]. Similarly, Li et al. performed successful Watchman implantation in 21 patients based on 3D model printing (3DP). In this study, although all patients in both groups underwent successful device implantation, significant differences did occur. After the occlusion, TOE showed that three patients in the control group had mild residual shunting (two patients with a 2 mm residual shunt, one patient with a 4 mm residual shunt). No residual shunt was observed in the 3DP group. The procedure times, contrast agent volumes, and costs were 96.4 ± 12.5 vs. 101.2 ± 13.6 min, 22.6 ± 3.0 vs. 26.9 ± 6.2 mL, and 12,676.1 vs. 12,088.6 USD for the 3DP and control groups, respectively. Compared with the control group, the radiographic exposure was significantly reduced in the 3DP group (561.4 ± 25.3 vs. 651.6 ± 32.1 mGy, *p* = 0.05) [19].

Goiten et al. found that LAA printed 3D models were accurate for prediction of LAA device size for the Amulet device but not for the Watchman device. Two procedures were aborted due to mismatch between LAA and any Watchman device dimensions in which all three interventional cardiology physicians that were involved in the study predicted the failures using the printed 3D model. Although 3D prints were found to be more accurate for Amulet compared to Watchman, strong agreement among physicians was demonstrated for both devices (average intra-class correlation of 0.915 for Amulet and 0.816 for Watchman) [20].

## 2. Algorithmic Approach for the WATCHMAN Procedural

**Figure 1:**
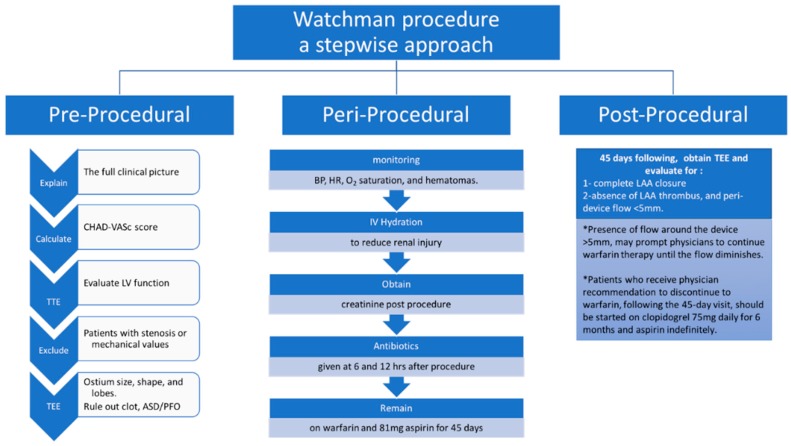
A stepwise approach for pre-, peri-, and post-procedure for the successful implantation of WATCHMAN device.

**Figure 2:**
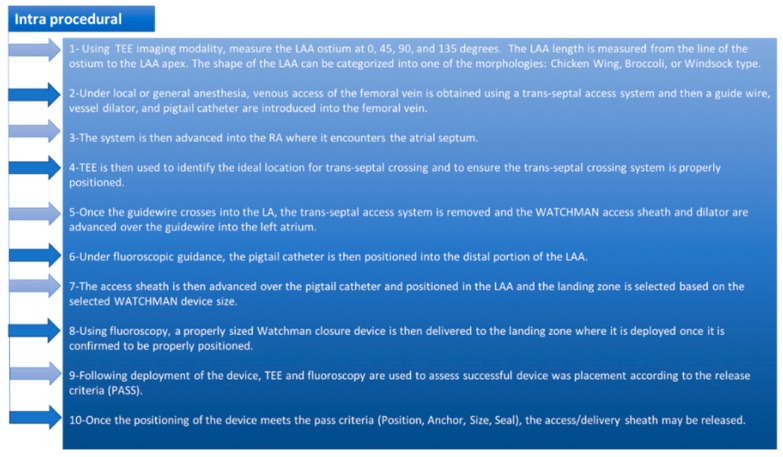
The WATCHMAN implant intra-procedural steps.

Here, we will provide an algorithmic approach including preprocedural, periprocedural, intraprocedural, and postprocedural. (Figure 1 and Figure 2) [21].

## 3. Pre-Procedural Assessment of the LAA

**Figure 3:**
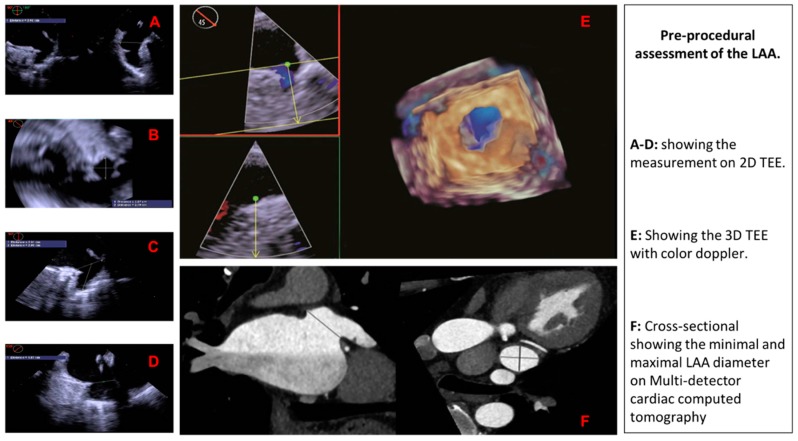
Pre-procedural assessment of the LAA using different imaging modalities.

Here, we will provide the pre-procedural assessment of the LAA on 2D TEE, 3D TEE, and MDCT (Figure 3).

## 4. Conclusions

Rotational three-dimensional transesophageal echocardiography is a more accurate alternative to two-dimensional transesophageal echocardiography in the assessment of the left atrial appendage morphology and has been the most commonly used imaging modality for sizing the left atrial appendage. Three-dimensional computed tomography has recently emerged as an imaging modality and it has showed exceptional accuracy when merged with three-dimensional printing technology. However, real-time three-dimensional transesophageal echocardiography may be considered the preferred imaging modality to assess left atrial appendage dimensions as it can provide accurate measurements without requiring radiation exposure or contrast administration (Table 6).

## Figures and Tables

**Table 1:** Advantages of 3D TEE vs. 2D TEE.

Author	Advantages of 3D TEE vs. 2D TEE	Study
Zhou et al.	-More accurate measuring of Landing zone and depth -More significant association between the closure device -Displaying cross-sectional images from any angle using Flexi Slice mode -Useful in displaying the LAA morphology after the occlusion device deployed -Visualizing any residual shunts around the entire device in one more view	[7]
Salzman et al.	-Producing (ADD) and (PDD) measurements of the LAA ostium -3D landing zone measurements demonstrated a higher reproducibility	[8]
Yosefi et al.	-3DTEE is a feasible, fast way to assess LAA morphology with similar accuracy to RT3DTEE and CT	[10]
Nucifora et al.	-RT3DTEE more significant agreement with the dimensions obtained from CT -RT3DTEE Provide accurate measurements without radiation exposure or contrast	[11]
Yosefi et al.	-RT3DTEE provides more accurate measurements of the maximal LAA orifice	[12]
Nakajima et al.	-Accurately visualize LAA morphological variations -Excellent correlation was found between full volume mode and zoom mode	[13]

**Table 2:** Literature review summary table for 3D TEE vs. 2D TEE in the preprocedural assessment of the left atrial appendage.

Ref	Author	Country	Date (mm/dd)	Objective	Study	Result/Outcome	Conclusion
[7]	Zhou et al.	China	01/17	To determine the clinical values of RT-3D TEE in the peri-procedure of LAA closure.	Observational study, of 38 patients conducted real-time 3D TEE (3D TEE) of the LAA for all subjects	-The landing zone dimension of LAA revealed by 2D TEE, showed statistical difference compared with the dimensions obtained from the 3D TEE -No statistical difference was noticed in the landing zone values of 3D TEE compared with that of X-ray -No statistical difference was noticed in the landing zone values of 3D TEE compared with that of X-ray	RT-3D TEE has better visualization of the LAA compared with 2D TEE.
[8]	Salzman et al.	Germany	07/17	To establish measurements based on 3D TEE imaging that would be most helpful in achieving successful cardiovascular intervention	Retrospective study analyzed 55 patient who underwent LAA occlusion using Watchman	ADD) and perimeter-derived diameter (PDD) from 3D TEE can reduce intra-procedural recapture maneuvers, peridevice leakage, and device size changes compared with two-dimensional (2D) measurements.	3D ADD and PDD may help with reducing intraprocedural recapture maneuvers, device size changes, and peridevice leakage.
[10]	Yosefi et al.	Israel	01/16	Compared RT3DTEE and 2DTEE versus CT when measuring LAA dimensions	Prospective study of 30 patients compared RT 3D TEE and 2D TEE versus 64 slice CT for measuring LAA dimensions	No difference was found between LAA depth using RT 3D TEE (19.5 ± 2.3 mm) vs. CT (19.6 ± 2.3, P = NS) and 2D TEE (19.4 ± 2.2 mm) vs. CT (P = NS). However, RT 3D TEE (24.5 ± 4.7 mm) vs. CT (24.6 ± 5, P = NS) was more accurate in measuring maximal LAA diameter compared to 2D TEE (23.5 ± 3.9 mm) vs. CT (*P* < 0.01).	RT3DTEE provides more accurate measurements of the maximal LAA orifice than 2D TEE.
[11]	Nucifora et al.	Switzerland	09/11	The accuracy of the measurements obtained via 2DTEE and RT3DTEE were subsequently compared against measurements obtained via CT.	Prospective study of 137 patients who underwent 2DTEE, RT3DTEE, and CT to measure the dimensions of the LAA orifice	-Compared to CT, both 2DTEE and RT3DTEE underestimated LAA dimensions. -RT3DTEE was found to be in greater agreement with the dimensions obtained from CT as demonstrated by smaller bias and narrower limits of agreement with CT	RT3DTEE may be the preferable imaging modality to assess LAA dimensions.
[12]	Yosefi et al.	Israel	09/16	To validate the accuracy of Rotational 3DTEE versus RT3DTEE when assessing LAA	Prospective study of 41 patients who underwent a rotational 3D TEE	Rotational 3D TEE measurements of LAA were not statistically different from RT3DTEE and from 64-slice CT regarding Rotational 3D TEE is achieved by rotating the sagittal plane (in the green box, x-plane) 360° and allows for a faster method of achieving necessary LAA measurements.	Choosing the appropriate device size for LAA closure can be achieved by Rotational 3DTEE (“Yosefy rotation”).
[13]	Nakajima et al.	Japan	09/10	To determined if 3D TEE could accurately visualize LAA morphological variations	Prospective od 107 patients, 55 were in SR in whom 3DTEE images were obtained from full-volume mode imaging, and 52 were in Afib, zoom-mode imaging was used.	3D TEE proviced adequate 3D full volume images of all patients in NSR, whereas adequate images were obtained in 94.6% of patients with AF using zoom mode. Excellent correlation was found between full volume mode and zoom mode.	3D TEE is a reliable modality when evaluating LAA geometry and LAA characteristics.

**Table 3:** Advantages of CT when assessing the left atrial appendage.

Author	Advantages of CT	Study
Chow et al.	-Allows more accurate assessment of the LAA ostium and landing zone. -Allows higher appreciation for the morphology of the LAA and surrounding structures	[14]
Rawjani et al.	-device sizing by CT-derived mean diameter was in most agreement with the actual device implanted -Better in detection and avoidance of sizing error by 2D TOE	[15]
Wang et al.	-WATCHMAN device selection was 100% accurate when selected by CT imaging -Provides a comprehensive assessment for LAA which is accurate	[9]
Budge et al.	-Provides accurate sizing of LAA occlusion devices	[16]

**Table 4:** Literature review summary table of CT imaging in the preprocedural assessment of the left atrial appendage.

Ref	Author	Country	Date (mm/dd)	Objective	Study	Result/Outcome	Conclusion
[14]	Chow et al.	Denmark	06/17	To compare available LAA imaging and sizing modalities which lead to successful LAA closure	Retrospective, 67 patients who underwent preprocedural MSCT and 2D TEE for LAA closure device sizing from 2014 to 2016	MSCT resulted in correct LAA sizing in 83% of patients, whereas 2D TEE would have produced in only 57% proper sizing	CT derived PD mean diameter may be the optimal measurement for sizing ‘closed-ended’ devices (Amulet and WATCHMANFLX) whereas CT derived maximal diameter is more accurate for sizing ‘open-ended’ devices (WATCHMAN)
[15]	Rawjani et al.	Australia	12/17	To evaluate the use of CT, procedural safety, and outcomes for percutaneous LAA closure	A registry between July 2010 and December 2015 was prospectively established for individuals undergoing LAA closure	2D TEE sizing resulted in gross sizing errors in 3.4% of cases. 2D-TEE measurements resulted in device selection that was 3mm smaller than those from CT measurements	CT has excellent outcomes for procedural safety with absence of major residual leak
[9]	Wang et al.	USA	11/16	To determine the role of 3DCT guided planning for LAA occlusion on the early operator WATCHMAN learning curve	Prospective study studied 53 patients who underwent 2D TEE, 3D TEE, and 3D CT for Watchman device qualification and sizing	53 patients underwent successful device implantation. Compared with 2D and 3D TEE sizing, 3D CT maximal width of the LAA landing zone was larger (*p* ≤ 0.0001). Pearson correlation coefficient showed a significant difference when sizing by CT against TEE (*r* < 0.001)	3D CT is an excellent tool in advanced case planning for precise WATCHMAN device size selection in LAA closure procedures compared to standard 2D TEE
[16]	Budge et al.	USA	11/08	To compare multiple different imaging modalities to assess the morphology of the LAA in AF patients	Prospective study of 66 patients where measurement relationships of TEE to planar CT (CTp), CTp to 3D cardiac segmented CT (CTsg), and CTsg to TEE were compared	Similar to CTp, CTsg orifice values were usually slightly smaller than TEE for large orifices, and larger than TEE for smaller orifices. LAA orifice measurements among CTsg, CTp, and TEE are not interchangeable which is clinically significant because of the need of accurate sizing of LAA occlusion devices	CTsg, either alone or in conjunction with TEE measurements, could allow for more accurate initial device sizing

**Table 5:** Literature review summary table of CT 3D printing in the preprocedural assessment of the left atrial appendage.

Ref	Author	Country	Date (mm/dd)	Objective	Study	Result/Outcome	Conclusion
[17]	Hell et al.	Europe	11/17	To determine If using 3D-printed LAA models based on CT will permit accurate device sizing	Prospective study of 22 patients who underwent pre-procedure TEE and CT examinations in which a 3D printed model was created based on the CT images and CT measurements recorded.	-Implantation was successful in all patients -In 95% of the patients (21/22), predicted device size based on simulated implantation in the 3D model was equal to the device ultimately implanted. TEE would have undersized the device in 45% of the patients (10/22) and device compression determined in the 3D-CT model corresponded closely with compression upon implantation of Watchman device (*r* = 0.622, *p* = 0.003).	CT 3D-printing models may assist with device selection and the prediction of device compression.
[18]	Li et al.	China	03/17	To assess 3DP feasibility using CT for LAA closure	Prospective study for 42 patients were randomly split into 2 groups, one that had 3D LAA model printing and a control group. For the control group, device size was was based on TEE, cardiac CT angiogram, and intraoperative LAA angiography only	The diameter of the occlusion devices used in the 3DP group and control group were 27.6 ± 2.4 mm (21–33 mm) and 26.3 ± 3.4 mm (21–33 mm), respectively. TOE showed that the compression ratios of the occlusion devices were 19.7% ± 0.8% and 19.3 ± 1.0% (*p* = 0.05), respectively.	3DP enhance the work efficiency for LAA closure which is valuable for clinical application.
[19]	Goiten et al.	Israel	10/17	To determine the feasibility of MDCT when predicting the accurate size of device for LAA closure	Prospective study including 29 patients compared 3D LAA model printing for predicting occlusion device size based on pre-procedure CT scan Amplatzer Amulet (St. Jude Medical/Abbott) was deployed in 12 patients and the other 17 received the Watchman device	Two procedures were aborted due to mismatch between LAA and any Watchman device dimensions in which all three interventional cardiology physicians that were involved in the study predicted the failures using the printed 3D model According to Bland-Altman analysis, the average difference between the predicted Amulet size using the 3D LAA printed model and the inserted Amulet was 0.848 mm (95% limit of agreement (LOA): −4.215, 5.912). The average difference between the predicted Watchman size using the 3D print and the inserted Watchman was 0.956 mm (95% LOA: −6.534, 8.445)	LAA 3DP model is not accurate for prediction of LAA using WATCHMAN devi.

**Table 6:** Preprocedural imaging impact on predicting the correct size of the WATCHMAN device.

Imaging Modality	Impact on Implantation Success
2D TEE	Less accurate
3D TEE	Accurate without requiring radiation exposure or contrast administration
3D CT	Exceptional accuracy when merged with three-dimensional printing technology
